# Dexamethasone-induced hepatic biochemical and pathological damages are attenuated by *Cynodon dactylon* extract containing hexadecanoic acid via reduction of glucose uptake: *In vitro* and *in vivo* studies

**DOI:** 10.22038/ajp.2025.26623

**Published:** 2026

**Authors:** Sadaf Mottaghian, Ayshin Khalilarya, Hassan Malekinejad

**Affiliations:** 1 *Experimental & Applied Pharmaceutical Sciences Research Center, Urmia University of Medical Sciences, Urmia, Iran*; 2 *Department of Pharmacology and Toxicology, School of Pharmacy, Urmia University of Medical Sciences, Urmia, Iran*

**Keywords:** Dexamethasone, Hepatic injuries, Hypolipidemic effect, Glucose uptake

## Abstract

**Objective:**

The protective effects of ethanolic extract of *Cynodon dactylon* (CD) on dexamethasone (DEX)-induced hepatic injuries were investigated.

**Materials and Methods:**

Following GC-MS phytochemical analyses of the extract,* in vivo* studies were conducted in male rats which were grouped as: control, dexamethasone (8mg/kg, intraperitoneally), DEX+CD (100, 200 and 400 mg/kg, orally), CD 400 and DEX+metformin (MET, 300mg/kg, orally) for 15 days. The *in vitro* studies were performed in HepG2 cells that grouped as: Control, DEX (1 µM), DEX+CD (0.1, 0.5 and 1 mg/ml), CD 0.5 mg/ml, and DEX+MET (500 µM). Cell viability and animal weight changes were recorded. Following the study period, biochemical and histopathological analyses were conducted on hepatic tissue and HepG2 cells.

**Results:**

Findings revealed that CD extract does have concentration-dependent free radical-scavenging activity, and it possesses phenols and flavonoids contents. The most abundant compound of the CD extract was n-hexadecanoic acid. The DEX-induced hepatic functional enzymes and lactate dehydrogenase levels were significantly (p<0.05) reduced in both models. Moreover, the DEX-induced oxidative stress was improved by CD extract (45%). The DEX-elevated blood glucose (20%) and triglycerides (60%) were reduced in CD-treated animals. The DEX-generated hepatic macrovesicles and lipid droplets in HepG2 cells were reduced.

**Conclusion:**

These results suggest that CD extract could be an effective compound for protection from DEX-induced hepatic injuries.

## Introduction

Drug-induced liver injury (DILI) is a worldwide concern and may lead to severe liver disease. Most cases of DILI are benign and improve after drug withdrawal. Adverse drug reactions (ADR) may result in discontinuance of the agent and hospitalization (Licata 2016). The liver is a prime target for medication-induced damages. A large group of medications can induce liver damage including: anesthetics, anticancers, antibiotics, anti-tuberculosis agents, anti-virals, and cardiac medications. DILI at the same time may be the result of immune-mediated mechanisms. Different types of drugs can cause hepatic injuries and they can also be interconnected; for instance, the hepatocyte destruction due to direct drug toxicity can be enhanced by the subsequent inflammatory reaction (Bessone et al. 2018). Produced bioactive intermediate mediators in inflammation phase , may interact with cellular organelles (e.g. mitochondria) leading to hepatocyte dysfunction and cell death (Wójcik et al. 2020). These toxic intermediate agents are relatively inactivated via glucuronic acid-, glutathione- or sulfa-conjugation in phase II reactions. Depletion or deficiency of the above-mentioned compounds responsible for this phase, may result in toxic metabolites accumulation (David et al. 2010). Inhibition of the mitochondrial respiratory chain is one of the circumstances during DILI, causing elevation in reactive oxygen species (ROS) and depletion of adenosine triphosphate (ATP) (Villanueva et al. 2021). ROS generation, ATP depletion and mitochondrial dysfunction may combine to induce intracellular damage. All of the above processes lead to hepatocyte apoptosis which requires energy (ATP) and may not be available due to mitochondrial dysfunction and depletion of ATP stores. In this case, liver cell death occurs via the necrotic pathway which exacerbates liver inflammation (Zhang et al. 2019). 

Corticosteroids are a class of steroid hormones released by the adrenal cortex, which includes glucocorticoids and mineralocorticoids. Glucocorticoids regulate diverse cellular functions including development, homeostasis, metabolism, cognition and inflammation. Due to their profound immune-modulatory actions, glucocorticoids are one of the most widely prescribed drugs. Unfortunately, the therapeutic benefits of glucocorticoids are limited by the adverse effects that are associated with high dose (used in the treatment of systemic vasculitis and systemic lupus erythematous) and long-term use. These side effects include osteoporosis, skin atrophy, diabetes, abdominal obesity, glaucoma, cataracts, avascular necrosis and infection, growth retardation, and hypertension. Furthermore, patients on long-term glucocorticoid therapy develop tissue-specific glucocorticoid resistance (Ramamoorthy and Cidlowski 2016). Since many of these adaptations are catabolic in nature, hence prolonged exposure to glucocorticoids can ultimately be detrimental (Meduri and Chrousos 2020). 

Glucocorticoids increase the risk of gastritis, gastric ulcer formation, and GI bleeding. Prolonged usage of GCs can lead to excessive deposition of lipids. 

Triglycerides (TG) are the main component of lipids that are contained in hepatic lipid droplets in liver resulting in non-alcoholic fatty liver disease (NAFLD). 

Among many side effects of GCs, insulin resistance with disturbances in glucose/insulin homeostasis and increased deposition of lipids is also important. Lipid deposition pathways stimulated by excess GC include elevated blood glucose levels due to stimulation of GC-induced gluconeogenesis, elevated glucose and insulin levels and stimulation of adipogenesis accomplished by the GCs themselves, as well as increased fat storage and stimulated release of free fatty acids upon absorption by the liver (Rahimi et al. 2020). Pathways that decrease hepatic lipids and are affected by GCs include a modest stimulation of very low-density lipoprotein synthesis and secretion into the circulation and inhibition of β-oxidation of fatty acids.

*Cynodon* *dactylon* as a medicinal plant contains various potentially bioactive compounds (Kalimuthu et al. 2022). The English name of *Cynodon* *dactylon* is Bermuda grass (Ashokkumar et al. 2013) and it belongs to the family Poaceae. It is native to East Africa, Asia, Australia and southern Europe. *C. dactylon* is a weed that has been shown to have several potential medicinal properties (Das et al. 2019). The extract of *C. dactylon* leaf has been reported to have anti diabetic (Putra et al. 2019), antioxidant and hypolipidemic effects (Marappan and Subramaniyan 2012). The aqueous extract of *C. dactylon *rhizome is used as a diuretic and anti-emetic, and against dysentery (Ashokkumar et al. 2013). The plant extract also has significant application in the treatment of dropsy and secondary syphilis (Pandey and Mishra 2019), wounds (Biswas et al. 2017), and inflammations (Alex et al. 2023). In a recent study, it was reported that *C. dactylon* extract was effective against bacterial pathogens and fungi (Savadi et al. 2020). The plant contains proteins, carbohydrates, minerals and other compounds like terpenoids, vitamin C, palmitic acid and alkaloids (Sangeetha and Baskaran 2010). Green grass contains (dry matter basis) 10.47% crude protein, 28.17% fiber and 11.75% of total ash (Pandey and Mishra 2019). Other important phyto-constituents reported from this plant were flavonoids: apigenin, luteolin, orientin and vitexin (Biswas et al. 2017); carotenoids: beta- carotene, neoxanthin, violaxanthin (Shabi et al. 2010), phenolics (Kumar et al. 2011), phytosterols, glycosides, saponins (Kumar et al. 2011) and volatile oils (Al-Snafi. 2016). 

With precise considering the molecular pathways of GCs-induced liver damages and the pharmacological capabilities of *C. dactylon* including its anti-inflammatory, anti-oxidant, hypolipidemic, anti-diabetic, anti-microbial and anti-atherosclerosis effects, we hypothesized that CD ethanolic extract may be medicinally useful in minimizing of GCs-induced hepatic injuries. To reach this goal, we performed various *in vitro* and *in vivo* studies using Hep-G2 cells as a known model of hepatic cells and rat animal model.

## Materials and Methods

### Plant collection and extraction

*Cynodon dactylon *plants were harvested from the agricultural grounds around the city of Urmia, Iran (June-August 2021). The identity of the harvested plants was confirmed (voucher number: HUPS: 338) at the department of pharmacognosy (School of Pharmacy, Urmia University of Medical Sciences, Urmia, Iran). The whole plant of *C. dactylon* was dried at room temperature and powdered to relatively fine powder using mechanical blender. Then, 250 g of dried powder was extracted by using Soxhlet extractor with 500 ml of ethanol (70%, v/v). The hydroalcoholic extract was concentrated using rotary vacuum evaporator (Heidolph, Schwabach, Germany) at 40°C. The final yield of extraction processes was 2.73%. 

### Radical scavenging activity measurement (DPPH assay)

To measure the capacity of extract in free radical scavenging, DPPH (2, 2-diphenyl-1-picryl-hydrazyl-hydrate) assay was performed according to a previous study (Kedare and Singh 2011). This method measures the ability of test compound in the decolorization of methanol solution of DPPH. Different concentrations of the extract were used to measure the hydrogen donating capability, which was characterized by changing the violet color of DPPH solution to yellow color. The absorbance of methanol solution of extract and DPPH mixture was measured spectrophotometrically at 517 nm. We used butylated hydroxytoluene (BHT) as a known scavenging agent. The following formula was used to calculate the DPPH radical scavenging capacity:

% DPPH radical scavenging activity = (A_C _− A_T_)/AC × 100

Where A_c_ is the absorbance of the control, and A_T_ is the absorbance of the extract. Then, % of inhibition was plotted against concentration of the extract. 

### Total phenol determination

To determine the phenolic contents of extract, the modified method of Folin-Ciocalteu was used (Nikolaeva et al. 2022). The extract was mixed with diluted Folin-Ciocalteu reagent (1:10 v/v water) and sodium carbonate (75 g/L). The samples were vortexed for 15 sec and remained for 20 min at 25ºC. The developed color absorbance was measured at 760 nm by using spectrophotometer (Bio Tek Instruments, EPOCH2TC, Inc. Highland Park, Winooski, VT, USA). Total phenol concentrations were expressed as gallic acid equivalent (GAE) (y = 0.1117x + 0.0229, R^2^ = 0.9997), mg of GA/g of dry extract.

### Total flavonoids measurement

Total flavonoid concentration was measured according to the previously published method (Gross 2004). Briefly, in this colorimetric assay, following the addition of sodium nitrate (150 ml, 5%) and distilled water (2.5 ml) to each sample/standard (0.5 ml) and 5 min incubation at room temperature, AlCl_3_ (0.3 ml, 10%) was added. After that, NaOH (1 ml, 0.001 M) and distilled water (0.5 ml) were added to all samples/standards and incubated at room temperature again for the next 15 min. Absorbance of mixed samples/standards was measured at 510 nm and total flavonoid concentration of the extract was expressed in terms of quercetin equivalent. 

### Analysis of extract (GC-MS analyses)

The ethanolic extract of *C. Dactylon *was analyzed by GC-MS to obtain the phytochemicals. An Agilent (5977B) GC-MS equipment along with column HP-5MS (length 30.0 m, diameter 0.25 mm, film thickness 0.25 μm) was used for analyses. In this study, 1 μl extract was injected into the GC-MS in split less mode at 290°C. The column oven temperature was held at 40°C for 2 min, then programmed at 10 °C/min until it reached to 290°C and hold for 5 min. Helium carrier gas was maintained at a flow rate of 1.0 ml/min. Total GC running time was 32 min.

### Experimental protocol – Study design

The current study was designed and performed in two models of *in vivo* and *in vitro. *

For *In vivo* studies, 35 male adult Wistar rats were used (170±20 g). The animals were randomly divided into seven groups each consisting of five rats. To induce dexamethasone (DEX)-related liver injury, 8 mg/Kg DEX was injected intraperitoneally (IP*, *0.4 ml final volume of injection/rat) (Kumar et al. 2015). Rats in group 1 (control) received 0.4 ml normal saline (IP) and 1 ml of dimethyl sulfoxide (DMSO) diluted with normal saline orally (for 15 days). In group 2 (DEX) rats received DEX by IP injection every day for the first week (7 days) and every other day for the second week (3 days). *Cynodon dactylon* extract was dissolved in DMSO and diluted with normal saline (final concentration of DMSO did not exceed 5%). Rats in groups 3 to 5, orally received 1 ml of *C. dactylon* extract solution (100, 200 and 400 mg/Kg B.W., respectively) after DEX injection (IP) in order to evaluate the protective effect of extract on the DEX-induced pathologic effects. Rats in group 6 only received 1 ml of the extract (400 mg/Kg, orally) for 15 days. Rats in group 7 (metformin) received both DEX and metformin (MET, 300 mg/Kg) for 15 days. Body weight of animals at the beginning of the experiment and at the end of the study was recorded. 

For *in vitro* studies, human HepG2 cells were cultured in Dulbecco’s Modified Eagle Medium DMEM containing 10% of fetal bovine serum and 1% penicillin/streptomycin (P/S) at 37°C and 5% CO_2_. Upon reaching a cell confluency of 80%, the cells were sub-cultured. The cells were treated and nominated as the following groups:

Cells in group 1 (control) were left untreated while cells in group 2 were treated with DEX (1 μM). Cells in groups 3 to 5 were simultaneously treated with DEX and *Cynodon dactylon* extract (0.1, 0.5 and 1 mg/ml, respectively). Cells in group 6 only received the extract (0.5 mg/ml) and cells in group 7 were treated with both DEX (1 μM) and MET (500 μM). After treatment, the cells were incubated for 24 hr and later, the cells and supernatants were removed for the pursuant analyses. 

Both parts of the current study were approved by the ethics committee of Urmia University of medical sciences, Urmia, Iran (IR.UMSU.REC. 1400.124 & IR.UMSU.REC. 1400.208).

### Tissue sampling and serum collection

Twenty-four hours after the last treatment, the rats were anesthetized with a cocktail of ketamine (80 mg/kg, IP) and xylazine (8 mg/kg, IP) and after collection of blood samples, animals were euthanized using a high dose of sodium pentobarbital (200 mg/kg, IP). The blood samples were collected through cardiac puncture and kept at room temperature for 15 min. To isolate the clear serum samples, the clotted blood samples were centrifuged at 1500 × g for 10 min and the isolated serum samples were preserved at -20°C for biochemical analyses. The liver specimens were dissected out and after removing any blood residues with chilled saline normal, were divided in two parts. The first part was snap-frozen in liquid nitrogen and kept at -80°C for further molecular experiments and the second part was kept in 10% formalin for histopathological examinations. 

### Biochemical parameters analysis

Alanine transaminase (ALT) and aspartate aminotransferase* (*AST) were determined in both supernatant of treated cells and in serum samples from *in vivo* studies by using commercial kits according to the manufacturer’s instructions (Darman Faraz Kave, Isfahan, Iran). Lactate dehydrogenase (LDH) level of serum and supernatant samples was determined using available kits and based on instructions of manufacturer (Pars Azmun, Tehran, Iran). Serum levels of lipid parameters including total cholesterol, total glycerides, Low Density Lipoprotein (LDL) and High Density Lipoprotein (HDL) were also measured using Pars Azmun (Tehran, Iran) commercial kits and their instructions. 

### Cell viability assay

To determine the effects of DEX and *Cynodon dactylon* extract treatment on cell viability, MTT colorimetric test was performed (Mosmann 1983). Briefly, HepG2 cells were seeded at a density of 8,000 cells per well in a 96-well plate (200 μl of medium) and incubated at 37°C for 24 hr. The cells were treated as mentioned in grouping section. After 24 hr treatment period, 20 μl of 3-(4,5-dimethylthiazol-2-yl)-2,5-diphenyltetrazolium bromide at 5 mg/mL in Phosphate Buffer Saline (PBS) was added to each well. The plate was incubated at 37 °C for 3 hr, and then the media was gently removed and 200 μl of DMSO was added to solubilize the formazan crystals. After thorough mixing, the plate was read on a spectrophotometer (BioTek Instruments, EPOCH2TC, Inc. Highland Park, Winooski, VT, USA) at 570 nm wavelength. Values were calculated using the following equation: 

Cell viability = (optical density of treated cells ∕ optical density of control cells) × 100

### Lipid peroxidation assay

To determine the lipid peroxidation rate, malondialdehyde (MDA) content of the samples was measured using the thiobarbituric acid (TBA) reaction. Briefly, 0.2-0.3 g of the liver samples were homogenized in KCl, and then, the mixture was centrifuged at 3000×*g *for 10 min. Thereafter, 0.2 ml of the supernatant/tissue homogenate was mixed with 1 ml phosphoric acid (1% V/V) and after vortex mixing, 1 ml of 6.7 g/L TBA was added to the samples. The samples were heated at 80°C for 45 min, and then chilled on ice. After adding 1 ml N-butanol, the samples were centrifuged at 3000×g for 10 min. The absorbance of the supernatant was measured spectrophotometrically at 532 nm and then the concentration of generated MDA was calculated according to the simultaneously prepared calibration curve using MDA standards. The amount of MDA is expressed as nMol per mg of sample’s protein. The protein content of the *in vivo* samples was assessed based on Lowery et al. method (Lowry et al. 1951).

### Measurement of total thiol molecules (TTM)

Total thiol molecules in the liver tissue and HepG2 cells supernatant samples were measured as described previously (Ranjbar et al. 2006). Briefly, the liver tissues were homogenized as mentioned above and centrifuged at 3000 × g for 10 min. Then, 200 μl of the supernatant of the tissue homogenate/cell supernatant was added to 600 μL Tris-EDTA buffer (Tris base 0.25 M, Ethylene diamine tetra-acetic acid: EDTA 20 mM, pH 8.2) and thereafter 40 μL 5.5’-Dithiobis-2-nitrobenzoic acid (10 mM in pure methanol) was added to a 10-ml glass test tube. The final volume of this mixture was made up to 4.0 ml by an extra addition of methanol. After 15 min incubation at room temperature, the samples were centrifuged at 3000 × g for 10 min and ultimately the absorbance of the supernatant was measured at 412 nm. The TTM capacity is expressed as nMol per mg of protein in samples. The protein content of the *in vivo* samples was measured according to the Lowry et al. method (Lowry et al. 1951).

### Measurement of serum/cell supernatant total antioxidant capacity (TAC)

To evaluate the total antioxidant capacity of serum/cell supernatant samples, the FRAP method was performed (Benzie and Strain 1999). To provide an acidic environment (pH 3.6) acetate buffer was used and the blue color produced due to the reduction of Fe^3+^ ions from the Fe^3+^- 2,4,6-Tris(2-pyridyl)-s-triazine (TPTZ) complex and converting them into ferrous (Fe^2+^) ions was assessed at 593 nm by a spectrophotometer. 

### Glucose uptake assay

HepG2 cells were seeded under above described condition and after 24 hr, were treated as expressed in experimental protocol using high glucose media (High glucose DMEM). After 48 hr of incubation, previous culture media was removed and replaced with low glucose media and insulin (100 nM) was added (Villanueva et al. 2021). HepG2 cells were incubated for about 30 min and glucose consumption was analyzed using commercial kit (Darman Faraz Kave, Isfahan, Iran).

### Histopathological examinations

The formalin-fixed and paraffin-embedded liver samples were subjected to hematoxylin and eosin (H&E) staining. The stained samples were examined under a light microscope (Nikon, Tokyo, Japan) and the DEX-related injuries were scored based on a simple 4-scale grading system (from 0 to 3) (Krishna 2021). The above mentioned scaling system was used for determining the proportion of hepatocytes with steatotic vacuoles. In grade 0, lipid droplets are only found in 5% of the hepatocytes. On the other hand, grade 1 steatosis refer to less than 33% of steatotic hepatocytes and in grade 2 and 3, lipid macrovesicles are detected in at least 33% or 66% of the hepatocytes, respectively.

### Oil red O staining

To analyze the lipid accumulation in the DEX-exposed HepG2 cells and to explore any potential protective effects of *Cynodon dactylon* extract, lipid droplets were stained by using oil red O staining kit (Asia Pajohesh, Amol, Iran). The scaling system was as same as that mentioned in the hepatic steatosis grading. 

### Statistical analyses

GraphPad Prism (Version 7.0; GraphPad software Inc., San Diego, USA) was used for statistical analyses. Results are presented as mean ± standard deviation. The comparisons among groups were made by analysis of variance (ANOVA) followed by Bonferroni *post hoc* test. Any changes in the body weight of animals before and after study were compared using paired T-test. A p value <0.05 was considered significant.

## Results

### Antioxidant capacity and phytochemicals of Cynodon dactylon ethanolic extract

To show the antioxidant capacity of *Cynodon dactylon* ethanolic extract, the free radical scavenging power (DPPH), total phenol content, total flavonoid concentration and ferric reducing antioxidant power for various concentrations of ethanolic extract were measured. All measured items indicate that the extract demonstrates a concentration-dependent anti-oxidant effects as reflected clearly in TAC assessment (Table 1). 

**Figure 1:**
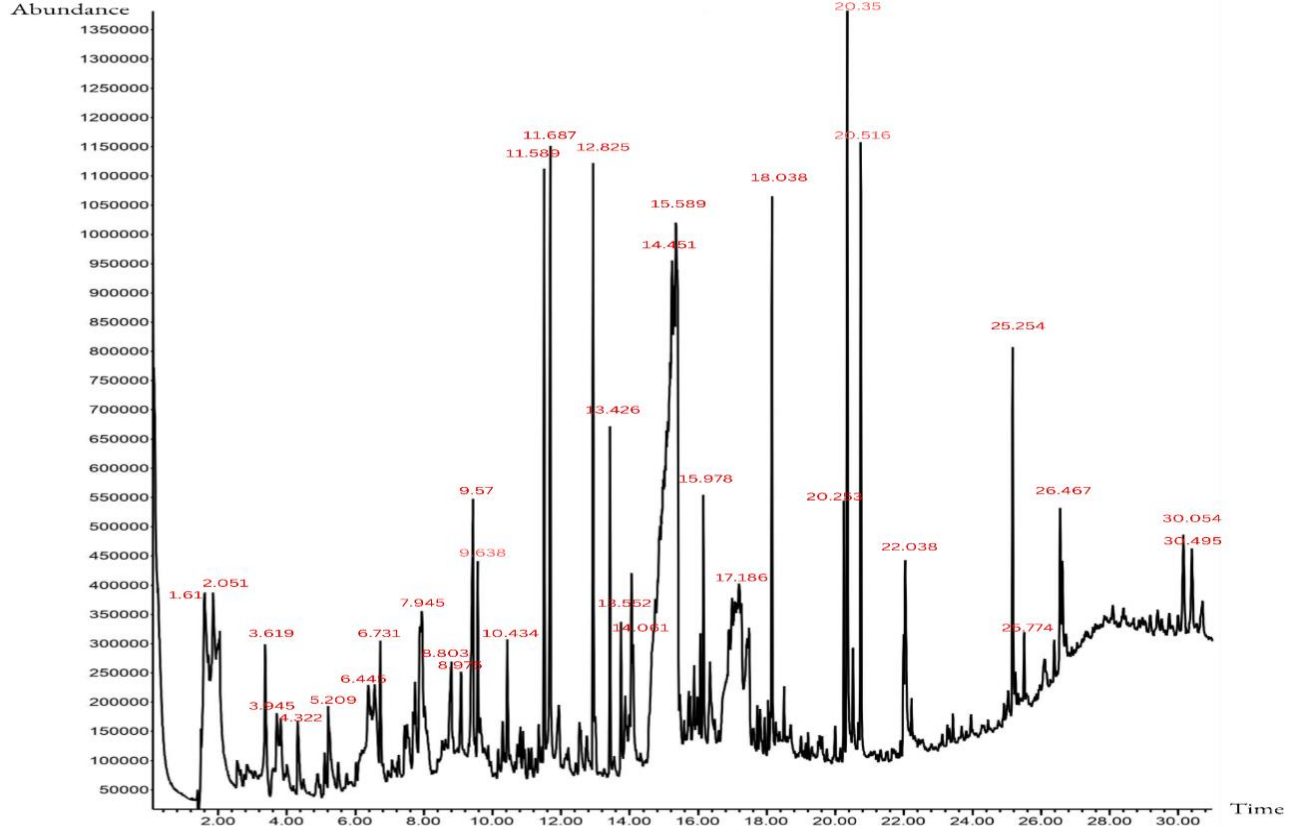
GC-MS chromatogram for ethanolic extract of *Cynodon dactylon*

Extract analysis by GC-MS technique revealed that the most abundant compound is n-Hexadecanoic acid, followed by pyrimidine derivatives, oleic acid and phenols (Figure 1 and Table 2). 

**Table 1:** Antioxidant capacity of Cynodon dactylon extract (CDE) represented by DPPH scavenging activity, total phenolic content, total flavonoids and total antioxidant capacity.

**CDE concentration (mg/ml)**	**DPPH (% of DPPH scavenging activity)**	**Total phenol (mg GAE/mg CDE)**	**Total flavonoid (mg QCNE/mg CDE)**	**TAC Eq nMol/L (FeSO4)**
1.56	13.7±2.7	0.37±0.017	0.78±0.04	24.3±1.0
3.125	37.3±1.1	0.45±0.01	2.43±0.07	38.2±5.2
6.25	67.7±3.1	0.65±0.1	5.79±0.61	56.7±3.0
12.5	82.1±7.1	0.87±0.006	7.99±0.18	89.1±5.0
25	91.3±3.1	1.28±0.074	11.88±0.17	176.1±8.5

**Table 2:** GC-MS analysis of ethanolic extract of *Cynodon dactylon*

**Compound**	**Extracted Compound Name **	**Molecular Formula**	**RT(min)**	**% **
1	n-Hexadecanoic acid	C₁₆H₃₂O₂	20.35	21.6
2	5-Methoxypyrimidin-4-ol	C_5_H_6_N_2_O_2_	11.687	21.3
3	Oleic Acid	C_18_H_34_O_2_	22.038	9.6
4	2,6-dimethoxyphenol	C_8_H_10_O_3_	13.426	7.7
5	2-Methoxyphenol	C_7_H_8_O_2_	9.57	7.1
6	Dimethyl(octadecyloxy)propylsilane	C_23_H_50_OSi	14.061	6.9
7	Benzenepropanoic acid, 3,5-bis(1,1-dimethylethyl)-4-hydroxy-, methyl ester	C_14_H_20_O	20.253	5.7
8	4-Methoxy-6-methyl-6,7-dihydro-4H-furo[3,2-c]pyran	C_9_H_12_O_3_	9.638	5
9	β-phenyl-Benzenepropanoic acid	C_15_H_14_O_2_	20.516	4.8
10	Succinic acid, eicosyl 4-methylpent-2-yl ester	C_30_H_58_O_4_	15.978	3.8
11	4-hydroxy-3-methoxy benzoic acid	C_8_H_10_N_2_O	15.589	2.4
12	Methyl 2-[4-(acetyloxy)-3-methoxyphenyl]acetate	C_12_H_14_O_5_	18.038	2
13	2-hydroxybenzaldehyde	C₆H₄OH	13.552	1.8
14	1,2,4-Benzenetricarboxylic acid, 4-dodecyl dimethyl ester	C_23_H_34_O_6_	25.254	1.7
15	3-fluoro-Benzenemethanol	C_7_H_7_FO	12.825	1.2
16	1,4-Bis(trimethylsilyl)benzene	C_12_H_22_Si_2_	26.467	1.1
17	Tris(tert-butyldimethylsilyloxy)arsane	C_18_H_45_AsO_3_Si_3_	26.467	1.1
18	1-Methyl-1-isopropyl-1-silacyclobutane	C_9_H_20_Si	11.589	1
19	1-(3-n-Propoxyphenyl)-2-propanone oxime	C_11_H_13_NO_3_	14.451	0.9
20	Deca-methyl tetra-siloxane	C_10_H_30_O_3_Si_4_	30.054	0.3

### Cynodon dactylon ethanolic extract declined the serum level of liver enzymes, lactate dehydrogenase and total antioxidant power in DEX-exposed Hep-G2 cells and DEX-administered rats

Hepatocytes functional enzyme level and supernatant concentration of LDH as a biomarker of cell membrane damage were determined and the results showed elevation of both enzymes and LDH concentration in DEX-received cells. Co-treatment of the HepG2 cells with DEX and various concentrations of *Cynodon dactylon* extract resulted in remarkable decline of hepatocytes functional enzymes levels and LDH concentration. The lowered concentration of AST, ALT and LDH by *Cynodon dactylon* extract was not concentration-dependent as the maximum reduction level was found in the group of cells that were treated with 0.5 mg/ml concentration of CD (Medium concentration). MET as a reference agent was able to reduce the DEX-induced ALT and LDH but not AST. Interestingly, 0.5 mg/ml CD alone also elevated the mentioned enzymes and LDH level. CD in a concentration-dependent manner elevated the DEX-reduced antioxidant capacity in HepG2 cell’s supernatant (Table 3A). In *in vivo* rat model also the hepatic functional enzymes level in serum along with LDH and TAC were measured. The obtained results indicate that CD at the 200 mg/kg dose level could reduce the DEX-elevated AST, ALT and LDH concentrations. Although CD alone at 400 mg/kg dose level increased the AST and ALT concentrations when compared with the control group, no significant change was shown in LDH levels of serum. At the same time, the DEX-declined total antioxidant power was significantly enhanced by CD extract and MET administration. Metformin and CD extract at 200 mg/kg dose level enhanced the serum level of TAC. CD alone after two weeks enhanced the TAC level remarkably (Table 3B).

**Table 3:** Protective effects of *Cynodon dactylon* extract on: (A) the DEX-induced changes in ALT, AST, LDH and total antioxidant capacity of HepG2 cells and (B) on the DEX-elevated ALT, AST, LDH and reduced total antioxidant capacity in rats. Data are presented as mean ± SD; asterisks are indicating significant differences (p<0.05) between the control and the DEX and/or *Cynodon dactylon* extract-received groups and hashtags are representing significant differences between the non-treated and CD extract treated DEX-received groups.

**A**							
	**Control**	**DEX**	**DEX+CD** _0.1 mg/ml_	**DEX+CD** _0.5 mg/ml_	**DEX+CD ** _1 mg/ml_	**CD** _0.5 mg/ml_	**DEX+Met** _500 __M_
AST (U/L) 10.8±0.7 15.6±0.11^*^ 15.3±0.04 15.1±0.24 16.6±0.2 13.3±0.09^*^ 16.0±0.61 ALT (U/L) 79.9±8.7 159.7±4.2 ^*^ 153.5±2.8 125.5±3.3^#^ 149.9±2.3^#^ 102.0±5.7^*^ 138.5±8.4 ^#^ LDH (U/L) 181.1±5.3 202.7±7.9 ^*^ 104.2±4.5^#^ 112.2±6.8^#^ 195.5±11.3 163.2±22.6 178.7±3.4 ^#^ TAC Eq nMol/L (FeSO4) 101.2±2.2 81.0±1.3^*^ 121.5±1.8^#^ 141.1±6.1^#^ 141.7±11.2^#^ 121.5±2.7^*^ 101.2±1.6 ^#^ **B** **Control** **DEX**							
							
							
							
							
			**DEX+CD** _100 mg/ml_	**DEX+CD** _200 mg/ml_	**DEX+CD** _400 mg/ml_	**CD** _400 mg/ml_	**DEX+Met** **300 mg/ml** AST (U/L) 145.4±5.1 227.5±9 ^*^ 194.4±7 ^#^ 159±39 ^#^ 192.1±20 ^#^ 189.3±2.5 ^*^ 178±2^ #^ ALT (U/L) 134.4±2.6 225.3±3.6 ^*^ 201±3.2 ^#^ 162.3±2.2 ^#^ 187.8±6.7 ^#^ 194.4±8.3 ^*^ 169.1±3.8 ^#^ LDH (U/L) 380.9±6 591.5±11 ^*^ 624.1±0.9 ^#^ 426±41 ^#^ 524.5±43 362±53^ *^ 383.1±28 ^#^ TAC Eq nMol/L (FeSO4) 371.9±8.8 259.5±5.3 ^*^ 232.8±8.4 ^#^ 372.6±9.7 ^#^ 357.1±7.5 ^#^ 468.2±6.3 ^*^ 345.6±14.2^#^
							
							
							
							

### Effects of DEX and CD on viability of HepG2 cells and body weight of rats

**Figure 2:**
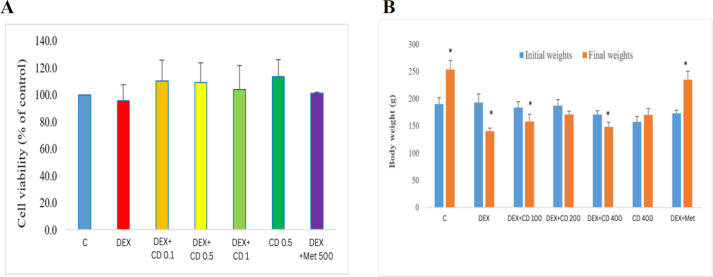
Effects of DEX and CDE on: **A**) viability of HepG2 cells and **B**) body weight changes. Data are presented as mean ± SD; asterisks are indicating significant differences between initial and final weights in each experimental group (p<0.05).

Exposure of HepG2 cells for 24 hr to DEX, CD extract, DEX plus CD/MET at given concentrations did not change significantly (p>0.05) the viability of cells (Figure 2A). 

Comparing the initial and final body weight of the animals in various experimental groups revealed that the DEX-received animals showed a significant weight loss, while CD extract slightly and MET profoundly reduced the DEX negative effects on body weight. MET administration at 500 mg/kg dose level resulted in a maximum weight gain in the DEX-received animals (Figure 2B). 

### CD ameliorated the DEX-induced oxidative stress in HepG2 cells and DEX-exposed rats

**Figure 3:**
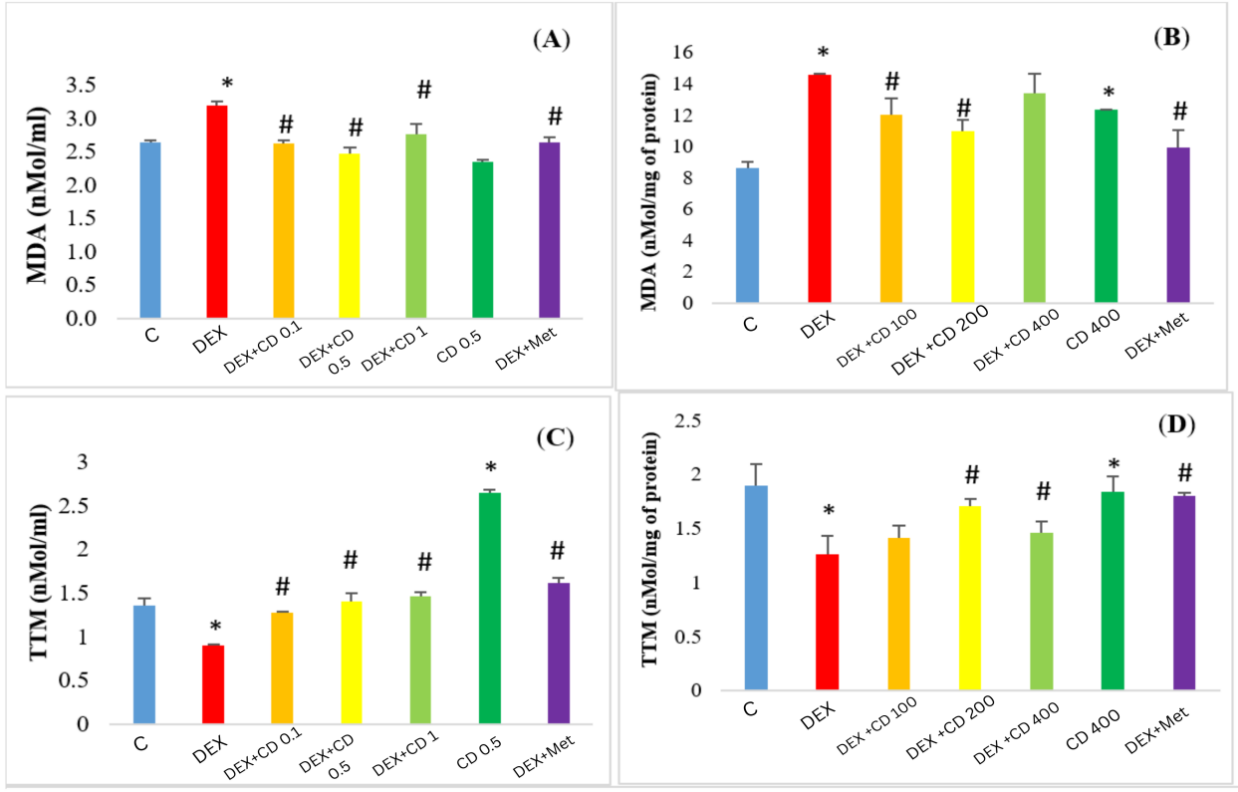
Effects of CD extract on DEX-induced oxidative stress: A) MDA generation and C) TTM content) in HepG2 cells and B) MDA generation and D) TTM concentration in the liver tissues of rats. Asterisks indicate significant differences (p<0.05) between the control and the DEX and/or CD-received groups and hashtags are representing significant differences between the non-treated and CD extract treated DEX-received groups.

Exposure of Hep-G2 cells and rats to DEX resulted in a significant elevation of MDA generation. At the same time, concurrent administration of DEX and CD in animals and HepG2 cells reduced the concentration of generated MDA. In both *in vivo* and *in vitro* approaches, MET attenuated remarkably the MDA generation and CD alone did not change the MDA content neither in the liver samples nor in HepG2 cells’ supernatant. The effect of CD on the MDA generation in both models was not dose/concentration-dependent as the maximum reduction in the MDA content was found at 200 mg/kg and 0.5 mg/ml of CD (Figure 3A and 3C). 

Total thiol molecules concentration was significantly (p<0.05) reduced in supernatant of HepG2 cells and the liver of animals that received DEX, while CD in HepG2 cells in a concentration-dependent and in rodent model in a dose-independent manner enhanced the DEX-reduced level of TTM. The highest level of TTM was found in the group of cells/animals which were exposed only against 0.5 mg/ml or 400 mg/kg of CD, respectively (Figure 3B and 3D). 

### CD reduced the glucose uptake by HepG2 cells, lowered the serum level of glucose and improved the lipid profile in DEX-exposed animals

**Figure 4:**
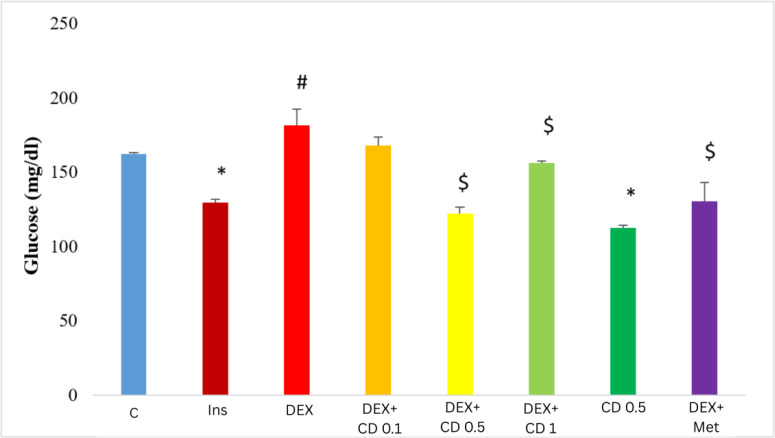
Effect of *Cynodon dactylon* extract on the DEX-induced insulin resistance in HEPG2 cells as demonstrated by glucose level in the supernatant of different experimental groups. Asterisks indicate significant differences (p < 0.05) between the control and the Insulin- and/or CDE-received groups, hashtag represents significant differences between the Insulin and DEX-received groups and $s show statistical significant differences between Insulin- and DEX-received groups which were treated with CD/MET.

Insulin treated HepG2 cells showed a significant decline in glucose concentration when compared with the corresponding control cells, while glucose level in the supernatant of DEX-received cells enhanced significantly (p<0.05). Those HepG2 cells which were co-exposed to DEX and CD, showed a decline of glucose level at 0.5 mg/ml CD concentration. CD at 0.1 mg/ml did not show statistically significant reduction of glucose. Metformin at the same time lowered the DEX-enhanced level of glucose (Figure 4). 

The blood glucose level in animals was measured and results made clear that the DEX-received group had higher glucose concentration than the control animals. Co-administration of DEX and CD lowered the glucose level in a dose-dependent manner. Metformin-treated animals showed a slight reduction of DEX-increased glucose concentration. CD alone did not change the blood glucose level remarkably (Table 4). Determination of serum level of lipid profile indices revealed that DEX exposure resulted in a slight and significant (p<0.05) increase of total cholesterol and LDL concentrations with no significant changes in HDL level. We recorded a remarkable elevation (2-fold) in total glycerides level in the DEX-received animals. CD administration on the other hand resulted in a significant reduction of the DEX-increased TC, TG and LDL concentrations in serum. Surprisingly, CD in the DEX-received animals reduced the serum level of HDL, while in intact CD-received animals the HDL level even exceeded the control animals (Table 4). 

**Table 4:** Effects of *Cynodon dactylon* extract on DEX-elevated blood glucose level and lipid profile. Asterisks indicate significant differences (p<0.05) between the control and the DEX and/or CD-received groups and hashtags represent significant differences between the DEX-received non-treated and CD extract-treated groups.

	**C**	**DEX**	**DEX+CD** _100 mg/ml_	**DEX+CD** _200 mg/ml_	**DEX+CD** _400 mg/ml_	**CD** _400 mg/ml_	**DEX+Met** _500 mg/ml_
Mean blood sugar (mg/dl)	112±1.4	131±6.9 ^*^	123±0.1^ #^	109±2.8^ #^	93±0.7 ^#^	101±4.9 ^*^	103±2.1 ^*^
Total Cholesterol (mg/dl)	64 ± 3	79±2 ^*^	61.5±1.5 ^#^	55±2.0 ^#^	50.5±0.5 ^#^	65±2	64.5 ± 1.5 ^#^
Total Glycerides (mg/dl)	89±6	177±6 ^*^	116±3.0 ^#^	108±5.5 ^#^	112.5±5.5 ^#^	74.5±3.5 ^*^	100± 3 ^#^
HDL (mg/dl)	40.5±0.5	39±2.0	33.5±1.5 ^#^	32.5±1.5 ^#^	34±3.0	43±0.5 ^*^	36.5 ± 1.5
LDL (mg/dl)	12.5±1.5	16.5±1.5^*^	10.5±0.5 ^#^	8.75±0.25 ^#^	12.5±0.5 ^#^	14±2.0	14.2 ± 1.7

### CD attenuated the DEX-induced lipid accumulation in HepG2 cells and in the liver of DEX-received rats

**Figure 5:**
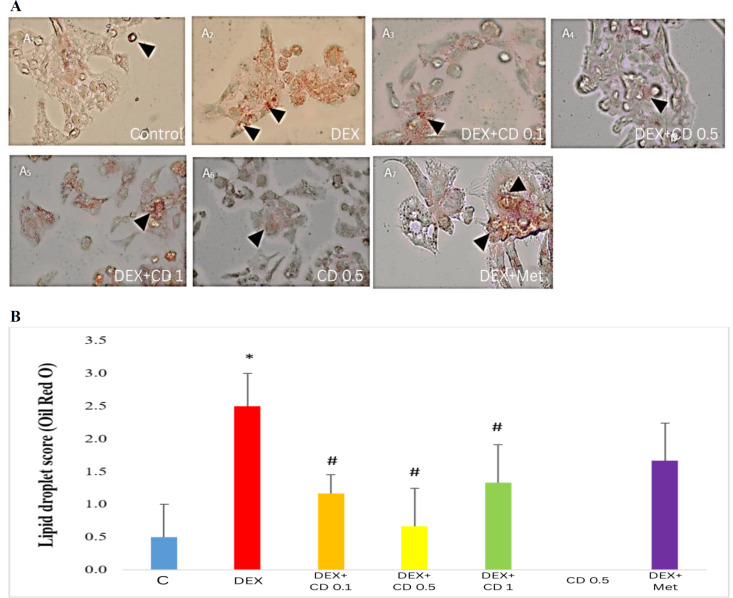
Protective effects of *Cynodon dactylon* extract (CDE) on DEX-induced lipid accumulation in HepG2 cells; A) Oil Red O staining, “A1 to A7” represent the control, DEX, DEX along with low, medium and high concentrations of CDE, CDE, DEX with metformin groups, respectively. The arrow heads are demonstrating lipid accumulation, (500 ×) and B) Lipid droplets scoring.

To analyze the lipid accumulation due to DEX exposure *in vitro* and *in vivo*, histological staining was performed. Our Oil Red O (ORO) staining in HepG2 cells revealed that following DEX exposure, remarkable accumulation of lipid droplets in cells’ cytoplasm are formed and presented in red color. The lowest grade of red color representing low lipid accumulation, was recorded in cells which co-treated with DEX and 0.5 mg/ml CD extract. Those cells which only exposed to CDE showed very tiny red color in ORO staining. Red color intensity grading analyses showed that the MET-received cells showed lipid droplets existence in moderate scale (Figure 5). 

**Figure 6:**
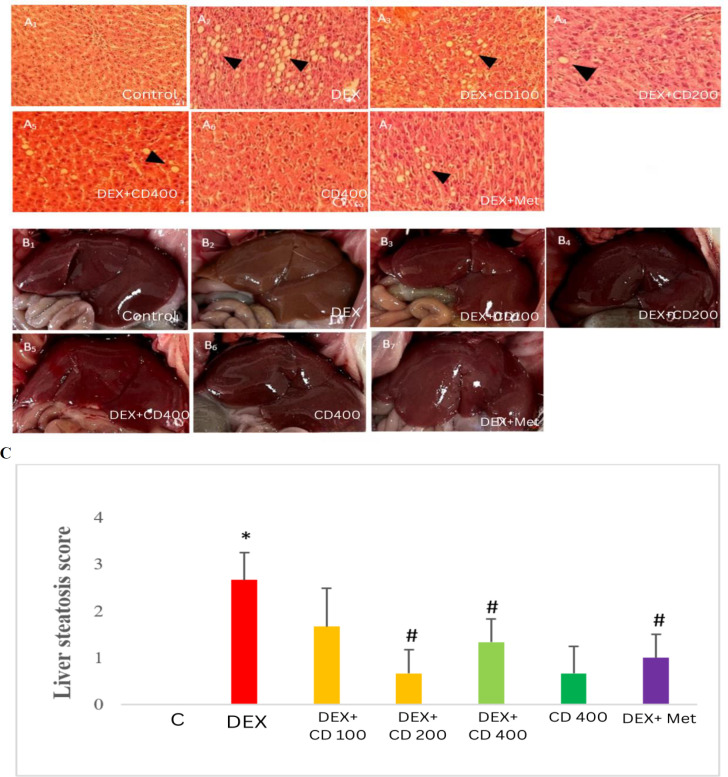
Effects of *Cynodon dactylon* extract (CDE) on DEX-induced lipid accumulation in the liver; (A) Hepatic histological changes were observed by H&E staining (400 ×), (B) Changes in liver color were observed at the end of treatment. “A1 to A7” or “B1 to B7” represented the control, DEX, DEX along with low, medium and high-dose levels of CDE, CDE, and DEX with metformin groups, respectively. The arrow heads demonstrate lipid accumulation, and (C) presents the liver steatosis score differences between various experimental groups.

At the same time, liver tissue staining showed very clear accumulation of lipid inside hepatocytes in the DEX-exposed group compared to control group. Co-administration of CDE and DEX resulted in a marked reduction of lipid droplets and also conversion of macrovesicles to microvesicles. CD administration in intact animals showed no considerable lipid accumulation in liver tissue. Metformin administration although lowered the lipid droplets but comparable lipid droplets in both macro and microvesicle forms were observed. Macroscopical record of the liver tissues revealed a pale hepatic color in the DEX-received animals compared to the control and other treated groups (Figure 6). 

## Discussion

The current study which has been performed in both *in vitro* and *in vivo* models, showed that DEX exposure in either models resulted in hepatotoxicity. The DEX-induced hepatotoxicity was characterized by elevation of hepatic functional enzymes concentration, LDH level, oxidative stress biomarkers, reduction of glucose uptake by HepG2 cells and elevation of blood level of glucose and triglycerides in the DEX-received rats, and ultimately with histopathological findings including accumulation of lipid droplets in hepatocytes. Moreover, CD with remarkable flavonoids contents, polyphenols, free radical scavenging capacity and total antioxidant power showed protective effects against DEX-induced hepatotoxicity in both study models. 

Despite worldwide prescription of DEX, there is a life threatening problem of misuse through the long time and high dose administration. Due to the biotransformation of DEX via hepatic cytochrome P450 3A isoenzymes, the liver is one of the main targets of the long time and high dose administration of DEX. DEX-induced hepatotoxicity was characterized by several biochemical and histopathological examination in both models of the current study. 

The first biomarkers to show the toxic effects of DEX in the liver were the high liver enzymes level (ALT and AST), which higher serum concentrations of them are indicate hepatocytes membrane damage. These enzymes act in gluconeogenesis by transferring of amino groups from aspartic acid or alanine. Hepatocellular injuries increase the activities of both enzymes and particularly ALT. The elevation of ALT and AST is not essentially indicating hepatocytes death. The elevated activity of ALT and AST may be related to either direct activation of enzymes by DEX or to the upregulation of their expression, which in turn results in activity elevation in plasma. At the same time due to intensifying effect of DEX on gluconeogenesis, which takes place dominantly by the two mentioned enzymes, elevation of ALT and AST concentration in the current study is clearly explainable. Another finding of the current study is that the DEX-received animals/HepG2 cells showed high glucose level in animals and lower glucose uptake (insulin resistance) in HepG2 cells. One of the important reason for hyperglycemia could be induction of both ALT isoforms in the hepatocytes due to DEX administration that results in glucose output elevation. Furthermore, it has been documented that the up-regulation of ALT at protein level in the liver (hepatocytes) is directly related to the insulin resistance, the situation which also occurs in diabetic patients (Qian et al. 2015). In addition of hyperglycemia, plasma lipid profile determination in the animal model of this study made clear that DEX administration at high dose resulted in elevation of triglycerides and total cholesterol. There are different mechanism for DEX-induced hyperlipidemia including: decreased lipoprotein lipase activity, increased hepatic lipogenesis, elevated very-low density lipoprotein formation in the gastrointestinal tract, declined the lecithin cholesterol acetyltransferases activity, and ultimately increased lipolysis in adipose tissues, which in turn elevates circulating fatty acids (Dourakis et al. 2002). 

Our findings indicate that the DEX-received animals showed a remarkable weight loss. This finding is in accordance with all previous studies indicating a glucocorticoids-induced atrophy in rodent model. Obviously, the main influential factor of body weight is skeletal muscle mass which is balanced by muscles protein synthesis and degradation. Previous studies showed that GCs either endogenously or exogenously cause muscle degradation via the ubiquitin proteasome pathway. In this pathway, ubiquitin ligase activation results in muscle protein degradation. Glucocorticoids-induced upregulation in the expression of Atrogin-1, muscle ring finger 1 (MuRF1) as main players of muscle atrophy has been documented (Yoshioka et al. 2019). Another influential factor in the DEX-induced muscle atrophy is up- or downregulation of different miRNAs. The up-regulation of miRNA-1, -322, -351 and -503 and downregulation of miRNA-147 and -708 under DEX-exposure in murine C2C12 myoblast cells have been shown (Shen et al. 2013). In addition of DEX-mediated gene expression pathway, there are studies indicating that DEX-induced oxidative stress is acting as another important pathway in the muscle atrophy. 

An imbalance between oxidant and antioxidant, which has been clearly demonstrated in the current study with DEX-increased MDA generation and glutathione depletion, results in a remarkable acceleration of protein catabolism and depression of protein synthesis (Powers et al. 2007). DEX-induced oxidative stress was recorded in both models of study as it has been reported already in rodent *in vivo* model as well as in various cells including adipocytes, osteoblastic, and pancreatic cells (Houstis et al. 2006). Induction of the ROS generation under DEX-administration also results in the upregulation of ubiquitin ligase, which in turn stimulates the degradation of insulin receptor substate-1, explaining a close relationship between the induced oxidative stress, body weight loss, hyperglycemia in *in vivo* studies and the increased MDA and reduced TTM concentration along with the sharp reduced glucose uptake in HepG2 cells (Uchida et al. 2018). To explain the cellular events following DEX administration that results in oxidative stress, increase of mitochondrial membrane potential and mitochondrial oxidation have been documented. The mentioned factors in turn result in enhancing the cellular metabolic rate and sequentially generation of reactive radical molecules (Bjelaković et al. 2007). There are increasing number of reports indicating that DEX at high dose and excessive use results in hyperlipidemia, which are confirmed by our findings.

 One of the important target organs for adverse effects of DEX is the liver. Our results showed macroscopically a pale liver feature compared with the corresponding control samples. Exactly in this regard not only hepatic functional enzymes elevated in the DEX-received animals but histopathological studies in both models of study showed microvesicular and macrovesicular fatty changes. The mentioned fatty changes following DEX-exposure were further confirmed with oil red O staining and demonstrating a prominent accumulation of lipid droplets in the cytoplasm of HepG2 cells. Most of the previous reports claimed that the accumulated lipids inside the hepatocytes are triglycerides and the well-known reasons for this accumulation related to the suppression of lipolysis and fatty acids β-oxidation in one hand and in the other hand a remarkable enhancement of the uptake of fatty acids (Jiao et al. 2020). In this study, following demonstration of DEX-induced impact in both *in vivo* and *in vitro* models, the second part of study devoted to clarify any beneficial and protective effects of ethanolic extract of CD on DEX-induced hepatic injuries. Our results clearly showed that CD not only in animal model reduced the DEX-induced hepatotoxicity, but in HepG2 cells also CD lowered the DEX-induced abnormalities. Several points regarding the hepatoprotective effects of CD must be taken in consideration: whether or not all DEX-induced hepatic damages were ameliorated by CD, what are the cellular and molecular pathways behind the CD protective effects, and the dose/concentration-dependency of CD related effects. 

Our findings showed that CD declined the DEX-elevated ALT and AST level significantly. Moreover, serum level of DEX-increased LDH as a common biomarker of organ damage was lowered by CD. All these findings are indicating that CD does have hepatoprotective and anti-inflammatory effects. Both mentioned effects of CD have been reported by previous researchers in various models of studies. For instance, the anti-inflammatory effects of CD on serotonin-, carrageenan-, and histamine-induced rat paw edema have been reported (Garg and Paliwal 2011). The phytochemical analyses showed that CD extract contains 21.6% n-Hexadecanoic acid as the most abundant compound. At the same time, the anti-inflammatory property of n-Hexadecanoic acid and oleic acid as the most abundant chemicals of CD has been previously reported (Ravi and Krishnan 2017; Santamarina et al. 2021). It seems that like as pathogenesis of hyperglycemia, induced by DEX, the hypoglycemic effects of CD are also at least partly related to the reduction of ALT activity. CD not only reduced the DEX-elevated blood glucose level but a marked reduction in DEX-increased lipids and in particular in triglyceride level was recorded. There are supporting data indicating that the ethanolic extract of CD, reduced the fasting blood sugar, cholesterol, triglyceride, and LDL in diabetic rats (Putra et al. 2019). The mentioned research team concluded that CD with stimulating the insulin secretion results in hypoglycemia and at the same time hypolipidemia as insulin is a potent inhibitor of lipolysis. Moreover, increasing the bile acid syntheses and its secretion may play a role in the reduction of plasma level of cholesterol and triglycerides. Antiglycation activity of CD has also been reported in *in vitro* model. The glucose uptake by CD-received cells was higher than that in non-treated cells as we reported in this study (Putra et al. 2019). Unlike the DEX-received animals that showed marked body weight loss, all other groups which concurrently received DEX and different dose levels of CD showed no significant body weight loss, suggesting a protective effects of CD from protein degradation due to DEX administration. According to previous report, it has been demonstrated that CD in diabetic rats by inhibition of hepatic glycogen degradation, reversing the gluconeogenesis and glycogenolysis prevented from hyperglycemia and from muscle wasting (Bharati et al. 2016). 

As stated early the DEX-induced oxidative stress likely is related to elevation of mitochondrial oxidation and sequentially free radical generation, which both results in lipid peroxidation and the glutathione resources depletion. Our results also revealed that CD ameliorated the DEX-induced oxidative stress by lowering the generated MDA level and replacing and/or preventing from thiol molecules depletion. The concentration-dependent free radical scavenging activity of CD was demonstrated in this study, supporting the antioxidant property of used CD. At the same time, other phytochemical analyses which have been performed in the current study showed that the ethanolic extract of CD does contain considerable amount of polyphenolic compounds along with high concentration of total flavonoids. The antioxidant effects of CD in the reduction of oxidative stress in diabetic rat and in complete Freund’s adjuvant-induced arthritis in rat model have been documented (Rai et al. 2010). More precisely, the enzymatic and non-enzymatic antioxidant activity of CD in the liver tissue has been reported (Devi et al. 2011).

Another important finding of the current study is that CD is able to remarkably diminish the microvesicular and macrovesicular fatty changes made by DEX high dose and excessive administration in the liver and in the hepatic HepG2 cells. Although there is report indicating the lipid lowering capability of CD, however no scientific study has shown lipid profile improvement following DEX-induced hyperlipidemia (Kaup et al. 2011). Moreover, our study for the first time showed that both *in vivo* and *in vitro* models could be used to monitor the DEX-induced hepatocytes injuries including lipid droplets accumulation. Additionally, based on the results of current study it would be possible to design the other pharmaceutical formulations such as CD phytosome or liposome for further studies to achieve the best practically useful formulations. It would be worth to note that simultaneous conduction of both models (*in vitro* and *in vivo*) need more expert researchers, which may be counted as limitation of these types of researches. 

Our results showed the hyperglycemia, hyperlipidemia, body weight loss, hepatic functional enzymes elevation and micro/macrovesicular fatty changes in DEX-received animals. Moreover, for the very first time results of this study explored the capacity of CD in the reduction of DEX-elevated blood glucose, lipids, serum level of ALT, AST and LDH along with ameliorative effects on DEX-induced body weight loss and hepatic lipid accumulation. Results of *in vitro* studies by using HepG2 cells supported all *in vivo* findings, suggesting a feasible and short time approach for such a drug/poison biosafety screening. 

## Data Availability

All data generated or analyzed during this study are included in this article.
